# Incidence of Exposure of Patients in the United States to Multiple Drugs for Which Pharmacogenomic Guidelines Are Available

**DOI:** 10.1371/journal.pone.0164972

**Published:** 2016-10-20

**Authors:** Matthias Samwald, Hong Xu, Kathrin Blagec, Philip E. Empey, Daniel C. Malone, Seid Mussa Ahmed, Patrick Ryan, Sebastian Hofer, Richard D. Boyce

**Affiliations:** 1 Section for Artificial Intelligence and Decision Support; Center for Medical Statistics, Informatics, and Intelligent Systems, Medical University of Vienna, Vienna, Austria; 2 Department of Pharmacy and Therapeutics, School of Pharmacy, University of Pittsburgh, Pittsburgh, Pennsylvania, United States of America; 3 College of Pharmacy, University of Arizona, Tucson, Arizona, United States of America; 4 Department of Pharmacy, College of public health and medical sciences, Jimma University, Jimma, Ethiopia; 5 Janssen Research and Development, Titusville, New Jersey, United States of America; 6 Observational Health Data Sciences and Informatics, New York, New York, United States of America; 7 Department of Biomedical Informatics, University of Pittsburgh, Pittsburgh, Pennsylvania, United States of America; Case Western Reserve University, UNITED STATES

## Abstract

Pre-emptive pharmacogenomic (PGx) testing of a panel of genes may be easier to implement and more cost-effective than reactive pharmacogenomic testing if a sufficient number of medications are covered by a single test and future medication exposure can be anticipated. We analysed the incidence of exposure of individual patients in the United States to multiple drugs for which pharmacogenomic guidelines are available (PGx drugs) within a selected four-year period (2009–2012) in order to identify and quantify the incidence of pharmacotherapy in a nation-wide patient population that could be impacted by pre-emptive PGx testing based on currently available clinical guidelines. In total, 73 024 095 patient records from private insurance, Medicare Supplemental and Medicaid were included. Patients enrolled in Medicare Supplemental age > = 65 or Medicaid age 40–64 had the highest incidence of PGx drug use, with approximately half of the patients receiving at least one PGx drug during the 4 year period and one fourth to one third of patients receiving two or more PGx drugs. These data suggest that exposure to multiple PGx drugs is common and that it may be beneficial to implement wide-scale pre-emptive genomic testing. Future work should therefore concentrate on investigating the cost-effectiveness of multiplexed pre-emptive testing strategies.

## Introduction

Ineffective medicinal treatments and drug-associated adverse events place a significant burden on modern healthcare systems [1]. Pharmacogenomic testing of patients prior to treatment initiation might help address these issues by tailoring pharmacotherapy to individual patient needs. Unfortunately, a current barrier to the widespread adoption of pharmacogenomic testing is the lack of information on how to implement it in an efficient and economic manner within clinical workflows [2,3]. Among potential implementation scenarios, “pre-emptive” population-based pharmacogenomic testing is a promising strategy. In pre-emptive testing, a panel of pharmacogenomic markers is tested once, and the test results are stored to optimize drug treatment in later patient care [2]. Still, healthcare organizations will likely not adopt pre-emptive testing without a clearer understanding of the magnitude of its potential impact, as well as associated costs.

Several leading health systems that have launched pharmacogenomics initiatives have reported their institutional processes and key implementation metrics. **Table** 1 provides an overview of some of these approaches, including targeted drugs and the testing procedures that were employed.

**Table 1:** Examples of health systems that have launched pharmacogenomics initiatives.

Institution	Drug(s)	Gene(s)*	Testing Approach
Vanderbilt University Medical Center [4]	clopidogrel, simvastatin, warfarin, thiopurines, tacrolimus	CYP2C19, SLCO1B1, CYP2C9, VKORC1, TPMT, CYP3A5	Genotyping panel (Illumina Veracode ADME)
St. Jude Children’s Research Hospital [5,6]	thiopurines, codeine, oxycodone, tramadol, amitriptyline, ondansetron, fluoxetine, paroxetine, simavastatin, clopidogrel, tacrolimus, capecitabine, fluorouracil, atazanavir, irinotecan, belinostat	TPMT, CYP2D6, SLCO1B1, CYP2C19, CYP3A5, DPYD, UGT1A1	Genotyping panel (Affymetrix DMET Plus) and a CYP2D6 copy number assay
University of Florida and Shands Hospital [7]	clopidogrel, thiopurines, codeine, tramadol, peg-interferon alpha	CYP2C19, TPMT, CYP2D6, IFNL3	Genotyping panel (Life Technologies Quant Studio OpenArray) and GenMark Dx (Carlsbad, CA)
University of Illinois at Chicago [8]	Warfarin, clopidogrel	CYP2C9, VKORC1, CYP4F2, CYP2C19	Genotyping panel (Genmark Dx eSensor)
Mayo Clinic [2]	abacavir, carbamazepine, thiopurines, interferon, citalopram, clopidogrel, escitalopram, warfarin, codeine, fluoxetine, fluvoxamine, paroxetine, tamoxifen, tramadol, venlafaxine, allopurinol, simvastatin	HLA-B, TPMT, IFNL3, CYP2C19, CYP2C9, VKORC1, CYP2D6, SLCO1B1	PGRNseq
University of Maryland [9]	clopidogrel	CYP2C19	Gene-specific test (Nanosphere Verigene)
Mount Sinai Medical Center [2]	Clopidogrel, warfarin, simvastatin, tricyclic antidepressants, selective serotonin reuptake inhibitors	CYP2C19, CYP2C9, VKORC1, SLCO1B1, CYP2D6	PGRNSeq

*CYP2C9: cytochrome P450 2C9, CYP2C19: cytochrome P450 2C19, CYP2D6: cytochrome P450 2D6, CYP4F2: cytochrome P450 4F2, SLCO1B1: solute carrier organic anion transporter 1B1, VKORC1: vitamin K epoxide reductase complex subunit 1, TPMT: thiopurine methyltransferase, HLA-B: major histocompatibility complex, class I, B, IFNL3: interferon, lambda 3.

For example, Vanderbilt University Medical Center’s PREDICT (Pharmacogenomic Resource for Enhanced Decisions in Care and Treatment) program [4] reports on the organization’s experience of genotyping 10 044 patients. The frequencies of actionable results (i.e., results that warrant a deviation from standard dosage and drug selection according to clinical guidelines) in 9 589 patients with complete genotype data based on the VeraCode ADME Core Panel (Illumina, San Diego, CA) that was used at the time were: CYP2C9/VKORC1-warfarin (70% of patients), CYP2C19-clopidogrel (28.5% of patients), SLCO1B1-simvastatin (25.7% of patients), CYP3A5-tacrolimus (23.5% of patients) and TPMT-thiopurines (9.1% of patients) [10]. Bush et al. examined the prevalence of actionable PGx variants based on sequencing data from about 5 000 subjects participating in the eMERGE-PGx program. They found that 96.19% of all samples had one or more actionable PGx variants [11].

At the time of this writing, few studies assessing the feasibility of large-scale pre-emptive pharmacogenomic testing are available. Dunnenberger et al. analyzed nationally representative outpatient drug prescriptions in the United States and found that a substantial number of prescriptions are for drugs with pharmacogenetic risk [2]. The PREDICT program published a cohort analysis of examining medication exposure, allele frequencies, and adverse event risk estimates in 52 942 patients at Vanderbilt University Medical Center [12]. In this population over a five year period, 65% were expected to receive at least one, and more than 10% were expected to receive at least four, medications for which evidence exists that the drug response is influenced by pharmacogenetics. Based on data on medication exposure and the event probability of six selected severe adverse drug reactions, they estimated the occurrence of 398 (95% CI: 225–583) severe adverse events that are potentially preventable by an effective pre-emptive testing program in their investigated patient cohort. Medications that posed the greatest risk included: clopidogrel (myocardial infarction, stroke, death), abacavir (hypersensitivity), azathioprine (leukopenia), simvastatin (myopathy), tamoxifen (breast cancer recurrence), and warfarin (bleeding). A pharmacoeconomic analysis was not performed, but the authors reported costs per adverse event estimates from three cost-effectiveness studies for abacavir ($121–$36 850, depending on severity of hypersensitivities), tamoxifen ($24 400–$56 521) and warfarin ($11 542).

Data from these studies suggest that pre-emptive pharmacogenomics testing might be most cost-effective when 1) a large number of different medications can be optimized by performing a single test and 2) future exposure to a multitude of these medications can be expected (e.g., elderly patients or patients with multiple morbidities).

The current study seeks to expand these analyses beyond a single academic medical center to quantify the incidence of pharmacotherapy in a nation-wide patient population that could be impacted by pre-emptive PGx testing based on currently available clinical guidelines in different hypothetical implementation scenarios. In particular, we aim to identify the fraction of patients across different age and insurance categories who had incident prescriptions of a multitude of different PGx drugs within a four-year time window. The purpose is to estimate the potential impact of pre-emptive PGx testing, and to provide the foundation for further in-depth cost-effectiveness analyses by large healthcare organisations and payers based on additional parameters such as estimated frequency and cost of adverse events, as well as costs of PGx testing.

## Materials and Methods

The study was conducted through the following analytical steps:

1. Identifying medications for which pharmacogenomic clinical guidelines are available (from here on called *PGx drugs*).
2. Analysing which PGx drugs were most frequently prescribed.
3. Describing the population of individuals exposed to PGx drugs by age and insurance coverage.
4. Determining the fraction of patients with incident prescriptions of a multitude of different PGx drugs within a 4 year period.
5. Comparing the results of different implementation models (pre-emptive vs. mixed reactive/pre-emptive approach) on the number of persons who would be eligible for genomic testing.
6. Estimating the frequency of high-risk drug–phenotype co-occurrences in the investigated populations

### Identification of medications with pharmacogenomic guidelines

Our analysis is based on well-established clinical guidelines for pharmacogenomic treatment optimisation from two organisations: the Clinical Pharmacogenetics Implementation Consortium (CPIC) [13,14] in the United States (US), and the Dutch Pharmacogenetics Working Group (DPWG) [15] in Europe.

The CPIC has published comprehensive reviews of the existing literature on specific drug-gene pairs and has authored guidelines on the clinical use of pharmacogenomic information. The CPIC guidelines are established in the field and have been endorsed by the American Society of Health Systems Pharmacists, incorporated into the US National Institute of Health’s Genetic Test Registry [16], and are indexed in PubMed as part of its Practice Guideline search tool.

The DPWG was formed by the Royal Dutch Pharmacist's Association in 2005 and has authored clinically applicable pharmacogenetic dosing recommendations for integration into an automated medical surveillance system in the Netherlands. The guidelines are developed through extensive systematic literature review.

We manually reviewed the pharmacogenomic treatment recommendations of both organisations in mid-2014 and compiled a unified list of PGx drugs mentioned in at least one of the guidelines.

To further refine the list of drugs to only those which have been widely established as important pharmacogenes and where testing is feasible, we compared the list of pharmacogenes covered in guidelines with genes that are covered by two consensus lists of important pharmacogenes–the PharmaADME Core Marker List [17] and PharmGKB VIP genes [18]–and seven panel-based pharmacogenomic assays to generate a ‘core PGx List’

### Patient data sources

Healthcare administrative claims data from three sources were used to establish population-level data on the frequency of prescription of pharmaceuticals for the PGx drugs:

1. Administrative claims data for a privately-insured population of over 100 million patients from multiple larger employers/payers in the US covering the years 2003 to 2013 (Truven MarketScan® Commercial Claims and Encounters, CCAE)
2. Administrative claims data for over 15 million Medicaid enrollees from multiple states in the US covering the years 2002 to 2012 (Truven MarketScan® Multi-state Medicaid)
3. Administrative claims for over 8 million US retirees with Medicare supplemental insurance paid by employers covering 2003 to 2013 (Truven MarketScan® Medicare Supplemental Beneficiaries)

In the US, commercial insurers (CCAE) include mostly working individuals and their families because health insurance is largely provided through employment. Because it represents a privately-insured population, relative to the US demographics, it underrepresents elderly and patients with lower socioeconomic status.

Medicare and Medicaid are government programs. Medicaid covers individuals who are economically disadvantaged. Qualifying patients for state Medicaid programs have lower socioeconomic status, and tend to be younger than the overall population. Medicare largely consists of older individuals over the age of 65 who are no longer working. In the dataset, only plans where both the Medicare-paid amounts and the employer-paid amounts were available and evident on the claims were selected. Because it presents patients who can afford supplemental coverage above and beyond the typical Medicare coverage afforded to all persons > 65 in US, our dataset tends to reflect a younger and healthier population with higher socioeconomic status than the overall Medicare population.

While the three MarketScan® databases are not directly nationally representative (without applying appropriate weights), they contain longitudinal data from a very large convenience sample of the US population and have been used in hundreds of population based studies. All datasets had been previously translated and loaded into a common data model and standard vocabulary as part of the Observational Medical Outcomes Partnership (OMOP) project [19,20]. Details on the decisions that were made by the OMOP project when translating and loading each dataset can be found in three publicly available mapping specification documents [21–23]. Version 4 of the common data model and standard vocabulary was used for all datasets.

A set of database queries was developed to conduct a cross-sectional evaluation of drug utilization across each dataset; database queries are available on GitHub [24]. This was possible because the standard vocabulary provided mappings to terms in the RxNorm terminology [25] for all medication claims contained in the administrative dataset described above. The three claims datasets were then queried using SQL Workbench/J (build 116) [26] using the a virtual computer instance provided by the Innovation in Medical Evidence Development and Surveillance (IMEDS) program [27]. The IMEDS laboratory is a secure Amazon Web Services Elastic Cloud Computing (EC2) image that provides approved researchers access to clinical research datasets that meet the US regulatory requirements for “de-identified” or regulatory “safe harbor.” The database queries are available online [28]. This study was reviewed by the University of Pittsburgh Institutional Review Board and found to include no involvement of human subjects.

### Prescription Drug Statistics

Statistics on incident claims for PGx drugs were generated within a selected four-year period (1/1/2009–12/31/2012). Incident claims were defined as administrative claims for a specific medication within the four-year period, without any previous claims for the medication prior to the start date of the four-year period. The rationale for focusing on incident use (i.e., excluding cases where a drug was already prescribed prior to the four-year period) instead of prevalent use (i.e., including all cases irrespective of prior drug use) was based on the assumption that treating physicians might not see enough value in optimizing treatment for patients that have already received a medication in the past without experiencing notable adverse events.

Topical preparations of PGx drugs were excluded because PGx guidelines are not generally relevant for topical therapies due to poor absorption. For each patient, the number of distinct PGx drug claims within the four-year period was calculated. Aggregate statistics were calculated for patient groups with specific ages at first time of PGx drug prescription. For the CCAE and Medicaid datasets, these age ranges (inclusive) were 0–13, 14–39 and 40–64 years; for the Medicare dataset, the age range was > = 65 years without an upper bound. These statistics were calculated for the core list of PGx drugs.

The aggregate statistics for prescription of medications were further stratified into two hypothetical, idealized scenarios: a ‘pre-emptive’ scenario, in which a pre-emptive genetic test for PGx genes would be conducted in the selected subpopulation at the start of the four-year period; and a mixed ‘reactive pre-emptive’ scenario, in which a genetic test panel would only be conducted at the time of first incident use of a PGx drug within the four-year time window. A single summary table was created to show how many different PGx drugs would be prescribed per tested patient in both scenarios, as well as the eight most prescribed PGx drugs for each dataset and age group.

### Estimation of phenotype distributions and clinical significance

#### Compiling haplotype frequencies and calculating the frequency of diplotypes in major population groups

Haplotype frequencies for all genes that are covered by at least one CPIC or DPWG guideline were compiled from studies cited in these guidelines [29–35]. These frequencies were used to estimate the frequencies of diplotypes (combinations of haplotypes) via Punnett squares. Punnett squares are a simple way to determine the probability of diplotypes by using a tabular representation of all potential haplotype combinations. Taking into account the partly large differences in haplotype frequencies between different ethnic groups, separate Punnett squares were created for major population groups (i.e. Caucasian, African / African American, Asian). Details on these Punnett squares are provided in S1 Table. The *1 haplotype was used as a “default” haplotype, indicating that none of the considered variants is present. For the calculations, the frequencies of *1 haplotypes was therefore imputed as 100%—(sum of all other haplotype frequencies in %) for all genes. An example of how diplotype frequencies were calculated is provided in S1 Text–Supplementary methods.

#### Estimating the distribution of drug metabolizing phenotypes in major ethnic population groups

Drug metabolizing phenotypes associated with diplotypes were inferred based on CPIC guidelines and, where CPIC guidelines were unavailable, on DPWG guidelines. For the majority of genes (i.e. CYP2C19, CYP2D9, CYP2D6, CYP3A5, DPYD, TPMT, UGT1A1), the following phenotype classification was used: Extensive metabolizer (EM), Poor metabolizer (PM), Intermediate metabolizer (IM) and Ultrarapid metabolizer (UM). For example, the CYP2C19*2/*3 diplotype (two loss-of-function alleles) was assigned to the phenotype “CYP2C19 Poor metabolizer”. For SLCO1B1, the phenotype categories “non-functional”, “intermediate function” and “low function” were used. For VKORC1, no phenotype classification was used since only one variant was considered. The diplotype-phenotype assignments for all genes included in this analysis are provided in S2 Table. For each gene and population group, the estimated overall frequencies of different drug metabolizing phenotypes were calculated by adding up the frequencies of the diplotypes assigned to the respective drug metabolizing phenotype. The estimated distributions of drug metabolizing phenotypes for major ethnic population groups can be found in S2 Table.

### Ethnic distribution in drug prescription datasets

The distribution of major population groups was derived for each dataset and age group. For the Medicaid dataset, the distribution of ethnic population groups was queried directly from the dataset. The categories “White” and “Black” reported in the dataset were assigned to the phenotype categories “Caucasian” and “African / African American”. The CCAE and Medicare Supplemental datasets did not contain data on population groups. Demographic statistics on these insurance populations were derived from an external source (i.e. the Kaiser Family Foundation [KFF], a US non-profit organization that publishes reports on health care issues) to estimate the distribution in the investigated cohorts [36,37]. A more detailed description on how ethnic distributions were inferred can be found in S1 Text.

### Clinical significance classification

The clinical significance of all drug-phenotype co-occurrences was categorized based on DPWG guidelines, which offer a unified categorisation scheme for most of the PGx drugs considered in this analysis (Table 2), as well as on CPIC priority levels, which are available for a subset of the considered PGx drugs. All of these assignments can be found in S4–S6 Tables. We focused our analysis on categories of higher clinical significance (i.e., DPWG classes C to F) and higher priority (i.e., CPIC priority level A).

**Table 2:** Classification of potential clinical effects observed in patients with risk phenotypes, based on DPWG guidelines.

Class	Clinical Effect
AA	Clinical effect (NS): no change or a non-significant change of clinical parameters. Kinetic effect (NS): no change or a non-significant change of kinetic parameters.
A	Minor clinical effect (S): QTc prolongation (<450 ms ♀, <470 ms ♂); QTc time increase < 60ms; INR increase < 4.5. Kinetic effect (S): significant change of kinetic parameters
B	Clinical effect (S): short-lived discomfort (< 48 hr) without permanent injury: e.g. reduced decrease in resting heart rate; reduction in exercise tachycardia; decreased pain relief from oxycodone; ADE resulting from increased bioavailability of atomoxetine (decreased appetite, insomnia, sleep disturbance, depressive mood, etc); neutropenia > 1.5x10^9^/l; leucopenia > 3.0x10^9^/l; thrombocytopenia > 75x10^9^/l; moderate diarrhea not affecting daily activities; reduced glucose increase following oral glucose tolerance test; muscle complaints creatine kinase <3 times normal upper limit
C	Clinical effect (S): long-standing discomfort (48–168 hr) without permanent injury e.g. failure of therapy with tricyclic antidepressants, atypical antipsychotic drugs; extrapyramidal side effects; parkinsonism; ADE resulting from increased bioavailability of tricyclic antidepressants, metoprolol, propafenone (central effects e.g. dizziness); increased INR 4.5–6.0; neutropenia 1.0–1.5x10^9^/l; leucopenia 2.0–3.0x10^9^/l; thrombocytopenia 50-75x10^9^/l; muscle complaints creatine kinase 3–10 times normal upper limit
D	Clinical effect (S): long-standing discomfort (> 168 hr), permanent symptom or invalidating injury e.g. failure of prophylaxis of atrial fibrillation; venous thromboembolism; decreased effect of clopidogrel on inhibition of platelet aggregation; ADE resulting from increased bioavailability of phenytoin; INR > 6.0; neutropenia 0.5–1.0x10^9^/l; leucopenia 1.0–2.0x10^9^/l; thrombocytopenia 25-50x10^9^/l; severe diarrhea; myopathy (muscle complaints creatine kinase ≥10 times normal upper limit)
E	Clinical effect (S): Failure of lifesaving therapy e.g. anticipated myelosuppression; prevention of breast cancer relapse; arrhythmia; neutropenia < 0.5x10^9^/l; leucopenia < 1.0x10^9^/l; thrombocytopenia < 25x10^9^/l; life-threatening complications from diarrhea; rhabdomyolysis
F	Clinical effect (S): death; arrhythmia; unanticipated myelosuppression

ADE: Adverse Drug Event. DPWG: Dutch Pharmacogenetics Working Group. NS: not statistically significant difference. S: statistically significant difference. INR: international normalized ratio. QTc: Corrected QT interval.

### Drug substances included in the estimation

Only a subset of the PGx drugs was included in this final step of our analysis (see Table 3). Drug substances with a clinical significance level A or B, and drug substances that could not be assigned a clinical significance level according to the DPWG classification were excluded. Furthermore, we decided to conduct two variants of the analysis; one excluding codeine, and one including codeine. The exclusion of codeine is likely to yield more accurate results since the drug is widely used as a low-dosed cough medicine, whereas PGx guidelines generally only apply to higher, analgesic dosages of codeine. A sufficiently reliable distinction of low-dose versus high-dose regimen of codeine was not deemed feasible with the datasets used. A detailed listing of the reasons for including / excluding drugs can be found in S1 Text–Supplementary methods.

**Table 3:** Overview of drugs for which CPIC or DPWG guidelines were available at the time of our analysis (mid-2014). Drugs and substances that we assigned to the ‘core list’ are listed separately. Only ‘core list’ substances were used for generating prescription statistics. Drug substances that were included in the estimation of the number of high-risk drug-phenotype co-occurrences are printed in bold.

Gene	Substances associated with gene in pharmacogenomic guidelines
Core list	
CYP2C19	**amitriptyline**^a^^,^^b^, **clomipramine**^a^^,^^b^, **clopidogrel**^a^^,^^b^, desipramine^a^, **doxepin**^b^, **imipramine**^a^^,^^b^, **nortriptyline**^a^^,^^b^, trimipramine^a^, citalopram^b^, escitalopram^b^, esomeprazole^b^, lansoprazole^b^, moclobemide^b^, omeprazole^b^, pantoprazole^b^, rabeprazole^b^, **sertraline**^b^, voriconazole^b^
CYP2C9	warfarin^a^, acenocoumarol^b^, glibenclamide^b^, gliclazide^b^, **glimepiride**^b^, phenprocoumon^b^, phenytoin^b^, tolbutamide^b^
CYP2D6	**amitriptyline**^a^^,^^b^, **clomipramine**^a^^,^^b^, codeine^a^^,^^b^, desipramine^a^, **doxepin**^a^^,^^b^, **imipramine**^a^^,^^b^, **nortriptyline**^a^^,^^b^, trimipramine^a^, aripiprazole^b^, atomoxetine ^b^, carvedilol ^b^, clozapine^b^, duloxetine^b^, flecainide^b^, flupenthixol^b^, **haloperidol**^b^, **metoprolol**^b^, mirtazapine^b^, olanzapine^b^, oxycodone^b^, **paroxetine**^b^, **propafenone**^b^, **risperidone**^b^, **tamoxifen**^b^, **tramadol**^b^, **venlafaxine**^b^, zuclopenthixol^b^
CYP3A5	tacrolimus^b^
DPYD	capecitabine^a^^,^^b^, fluorouracil^a^^,^^b^, tegafur^a^^,b^
TPMT	**azathioprine**^a^^,^^b^, **mercaptopurine**^a^^,^^b^, **thioguanine**^a^^,^^b^
UGT1A1	irinotecan^b^
SLCO1B1	simvastatin^a^
VKORC1	warfarin^a^, phenprocoumon^b^
Others (not included in drug prescription statistics)	
F5	estrogen-containing oral contraceptives^b^
HLA-B	abacavir^a^^,^^b^, allopurinol^a^, carbamazepine^a^, ribavirin^a^^,^^b^
IFNL3	peginterferon alfa-2a^a^, peginterferon alfa-2b^a^, ribavirin^a^^,^^b^

a: substance covered by CPIC guideline

b: substance covered by DPWG guideline. CPIC: Clinical Pharmacogenetics Implementation Consortium. DPWG: Dutch Pharmacogenetics Working Group.

### Estimating the number of high-risk drug-phenotype co-occurrences

Calculating the probability that a PGx drug is prescribed to a patient who has a drug metabolizing phenotype that puts the patient at risk for developing an adverse drug reaction if the drug is prescribed in standard dosage was performed as follows:

$$p\left(Rx\right|\mathrm{risk phenotype})=p\left(Rx\right)\times p\left(\mathrm{risk phenotype}\right|\mathrm{ethnicity})\times p(\mathrm{ethnicity})$$

where p(Rx) is the probability that a patient is prescribed a PGx drug in the observed time frame (see S3 Table), p(risk phenotype | ethnicity) is the estimated prevalence of risk phenotypes relevant to the respective PGx drug in the respective population group (see S2 Table) and p(ethnicity) is the prevalence of the ethnic population group in the respective dataset and age group (see S4–S6 Tables).

These calculations were performed for all PGx drug–risk phenotype combinations considered in this analysis, across all datasets (Medicaid, Medicare, CCAE), age groups, and ethnicities (Caucasian, African / African American, Asian). The results were summed up for each dataset, age group and clinical significance level. The calculations can be found in S4–S6 Tables. An exemplary calculation for the number of CYP2C19 poor metabolizer / codeine prescription co-occurrences in the Medicare dataset is provided in S1 Text.

## Results

Based on pharmacogenomic guidelines, we included 61 drugs in the analysis (Table 3). From these, 10 medications were associated with two different genes resulting in 72 different drug-gene interaction pairs (Table 3).

A total of 24 interaction pairs were derived from CPIC guidelines and 61 interaction pairs were derived from DPWG guidelines. Thirteen drug-gene interaction pairs were included in both guideline sources. We found that some of the genes covered by PGx guidelines were included in the majority of consensus lists and assays (e.g., CYP2C19, CYP2D6), while a small set of other genes were rarely included (e.g., “Others”—F5, HLA-B, IFNL3). From here on, we refer to genes and drugs that we found to be well-established based on this analysis as the *core list* of genes and drugs (Table 3).

### Pre-emptive testing

In total, 73 024 095 patient records were included in the analysis, of which 55.7% were associated with female patients. An overview of the results is shown in Table 4, with greater detail provided in several spreadsheets as supplementary material (S3–S6 Tables). The top prescribed PGx drugs were indicated for pain relief and cardiovascular conditions. Incident use of PGx drugs in the 0–13 year of age range was very low, with only 1.1% (CCAE) to 1.8% (Medicaid) receiving two or more different PGx drugs. Incident use of two or more PGx drugs increased with age, rising to 17.8% (CCAE age 40–64) and 32.8% (Medicaid age 40–64), respectively. In general, the utilisation of PGx drugs in the Medicaid dataset was significantly higher than in the CCAE dataset. Patients in the Medicare dataset (age > = 65) received a large number of PGx drugs; with 27.5% receiving two or more PGx drugs. Still, utilisation of medications that may benefit from genomic testing was lower in the Medicare dataset than in the patients of age 40–64 who were enrolled in Medicaid.

**Table 4:** Incidence of exposure to drugs for which pre-emptive pharmacogenomic testing is available.

Characteristic	CCAE (age 0–13)	CCAE (age 14–39)	CCAE (age 40–64)	Medicaid (age 0–13)	Medicaid (age 14–39)	Medicaid (age 40–64)	Medicare (age > = 65)
n	9 893 962	22 824 848	26 561 525	4 151 506	3 032 191	1 130 797	5 429 266
Female	48.1%	57.5%	54.8%	48.4%	69.3%	60.8%	55.2%
Age (median, mean)	6, 6.01	27, 26.3	51, 51.23	5, 5.14	21, 22.5	50, 50.57	72, 73.82
**Fraction of patients with incident use of a given minimum number of distinct PGx drugs within the observed four-year time window. First value for pre-emptive scenario, second value in brackets for 'reactive pre-emptive' scenario.**							
> = 1 drugs	11.2% (100.0%)	30.4% (100.0%)	42.2% (100.0%)	14.0% (100.0%)	40.2% (100.0%)	55.5% (100.0%)	50.6% (100.0%)
> = 2 drugs	1.1% (9.9%)	9.1% (30.0%)	17.8% (42.2%)	1.8% (12.1%)	15.3% (38.1%)	32.8% (59.0%)	27.5% (54.4%)
> = 3 drugs	0.2% (2.1%)	3.1% (10.2%)	7.5% (17.8%)	0.5% (3.3%)	6.5% (16.1%)	18.5% (33.1%)	13.8% (27.3%)
> = 4 drugs	0.1% (0.5%)	1.1% (3.8%)	3.1% (7.4%)	0.1% (0.9%)	2.9% (7.2%)	9.9% (17.8%)	6.4% (12.7%)
> = 5 drugs	0.0% (0.%)	0.4% (1.4%)	1.3% (3.0%)	0.0% (0.3%)	1.3% (3.2%)	5.0% (9.1%)	2.8% (5.5%)
> = 6 drugs	0.0% (0.0%)	0.2% (0.6%)	0.5% (1.2%)	0.0% (0.1%)	0.6% (1.5%)	2.4% (4.3%)	1.1% (2.3%)
**PGx drugs with most frequent incident use within four-year time window**							
Rank 1	Codeine (7.2%)	Codeine (9.4%)	Codeine (9.5%)	Codeine (8.8%)	Oxycodone (15.0%)	Oxycodone (15.8%)	Simvastatin (13.4%)
Rank 2	Lansoprazole (1.7%)	Oxycodone (7.8%)	Oxycodone (8.8%)	Lansoprazole (1.4%)	Codeine (10.6%)	Tramadol (13.8%)	Metoprolol (10.8%)
Rank 3	Omeprazole (0.6%)	Tramadol (4.0%)	Simvastatin (8.2%)	Risperidone (1.1%)	Tramadol (8.3%)	Omeprazole (10.9%)	Omeprazole (9.2%)
Rank 4	Atomoxetine (0.5%)	Sertraline (3.2%)	Omeprazole (6.2%)	Omeprazole (1.0%)	Citalopram (4.8%)	Simvastatin (9.6%)	Tramadol (8.5%)
Rank 5	Sertraline (0.5%)	Omeprazole (3.1%)	Tramadol (6.2%)	Oxycodone (0.7%)	Omeprazole (4.6%)	Citalopram (7.6%)	Codeine (8.2%)
Rank 6	Risperidone (0.4%)	Citalopram (2.9%)	Metoprolol (4.4%)	Atomoxetine (0.6%)	Sertraline (4.3%)	Metoprolol (7.3%)	Oxycodone (8.0%)
Rank 7	Oxycodone (0.3%)	Escitalopram (2.2%)	Citalopram (3.3%)	Sertraline (0.6%)	Aripiprazole (1.9%)	Codeine (7.1%)	Warfarin (5.6%)
Rank 8	Aripiprazole (0.2%)	Pantoprazole (1.3%)	Sertraline (2.8%)	Aripiprazole (0.5%)	Risperidone (1.8%)	Sertraline (4.4%)	Clopidogrel (5.1%)

CCAE: Truven MarketScan® Commercial Claims and Encounters dataset. PGx drugs: drugs for which pharmacogenomic guidelines are available.

### ‘Reactive pre-emptive’ testing

The results for the ‘reactive pre-emptive’ scenario differ significantly from the results of the purely pre-emptive scenario (Table 5 and S3 Table). Since the ‘reactive pre-emptive’ scenario is based on the assumption that the pre-emptive pharmacogenomic test is triggered when the first incident use of a PGx drug occurs, 100% of tested patients in this scenario receive at least one PGx drug. The fraction of tested patients receiving two or more different PGx drugs is also considerably greater in this scenario across all groups (Table 4). For example, in both the CCAE 14–39 and 40–64 groups, the fraction of patients receiving two or more PGx drugs is tripled compared to the pre-emptive scenario (rising from 9% to 31.1% and from 17.8% to 43.3%, respectively). In the Medicare > = 65 group the fraction is doubled (from 27.5% to 54.6%). The highest relative increase is seen in the youngest age group, e.g., a rise from 1.1% to 9.6% in the CCAE 0–13 group.

**Table 5:** Number of expected drug-phenotype co-occurrences of highest priority according to CPIC guidelines (CPIC level A) or high clinical significance according to DPWG guidelines (DPWG clinical significance classes C–F) within the observed four-year time window. PGx drugs included in all estimations: amitriptyline, azathioprine, clomipramine, clopidogrel, doxepin, glimepiride, haloperidol, imipramine, mercaptopurine, metoprolol, nortriptyline, paroxetine, propafenone, risperidone sertraline, tamoxifen, thioguanine, tramadol, venlafaxine. Estimations which additionally included codeine are shown for comparison. The rationale for including / excluding drug substances is described in the Methods section (‘Drug substances included in the estimation’).

	CCAE (age 0–13)	CCAE (age 14–39)	CCAE (age 40–64)	Medicaid (age 0–13)	Medicaid (age 14–39)	Medicaid (age 40–64)	Medicare (age > = 65)
**n**	9 893 962	22 824 848	26 561 525	4 151 506	3 032 191	1 130 797	5 429 266
**CPIC (excluding codeine)**							
**CPIC level A**	3 317 (0.0%)	53 341 (0.2%)	223 915 (0.4%)	1 919 (0.0%)	12 149 (0.4%)	11 252 (1.0%)	111 095 (2.0%)
**CPIC (including codeine)**							
**CPIC level A**	31 189 (0.3%)	137 906 (0.6%)	445 607 (0.8%)	15 370 (0.4%)	23 866 (0.8%)	14 183 (1.3%)	128 905 (2.4%)
**DPWG (excluding codeine)**							
DPWG class C	10 324 (0.1%)	155 461 (0.7%)	285 894 (1.1%)	9 225 (0.2%)	39 739 (1.3%)	27 680 (2.4%)	82 003(1.5%)
DPWG class D	2 840 (0.0%)	19 141 (0.1%)	84 057 (0.3%)	2 167 (0.1%)	4 728 (0.2%)	6 739 (0.6%)	41 927 (0.8%)
DPWG class E	352 (0.0%)	959 (0.0%)	8 463 (0.0%)	148 (0.0%)	109 (0.0%)	757 (0.1%)	2 786 (0.1%)
DPWG class F	265 (0.0%)	5 921 (0.0%)	120 556 (0.5%)	157 (0.0%)	1 361 (0.0%)	13 745 (1.2%)	91 346 (1.7%)
**Sum of DPWG class C—F**	13 781 (0.1%)	181 482 (0.8%)	605 534 (1.9%)	11 697 (0.3%)	59 694 (1.5%)	48 921 (4.3%)	218 062 (4.1%)
**DPWG (including codeine)** ^**a**^							
**Sum of DPWG class C—F**	41 653 (0.4%)	266 047 (1.2%)	598 302 (2.3%)	25 147 (0.6%)	57 654 (1.9%)	51 852 (4.6%)	235 871 (4.4%)

^a^ Detailed per-class estimates including codeine were omitted here; full data can be found in S7 Table. CPIC: Clinical Pharmacogenetics Implementation Consortium. DPWG: Dutch Pharmacogenetics Working Group. CCAE: Truven MarketScan® Commercial Claims and Encounters dataset.

**Fig 1:**
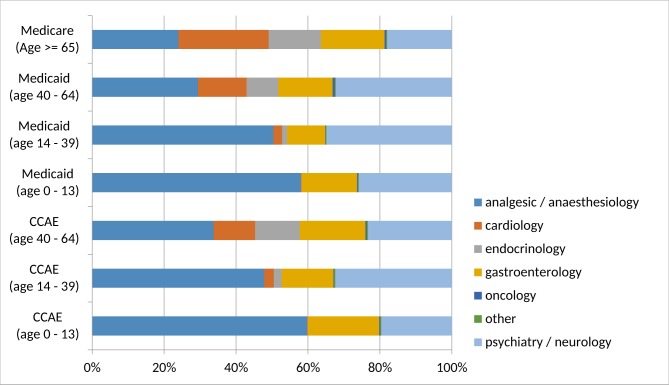
Distribution of incident use within four-year time window among therapeutic areas.

The relative distribution of PGx drugs among therapeutic areas is shown in Fig 1. Analgesics/anaesthesiology and psychiatry/neurology medications made up a large fraction of PGx drugs across all groups studied with cardiology and endocrinology medications becoming increasingly important with advanced age.

### Clinical significance

Table 5 presents the estimated fractions of patients with drug-phenotype co-occurrences that pose a risk for inducing significant adverse events for all investigated datasets, broken down by DPWG clinical significance levels (see Table 2 for the categorisation scheme) and CPIC priority level. When focusing on the estimates not including codeine, the greatest estimated overall fractions of high risk drug-phenotype co-occurrences across all clinical significance levels were found in the Medicaid 40–64 and the Medicare > = 65 group (4.3% and 4.1%, respectively). Our estimation of drug-phenotype co-occurrences assigned with the greatest clinical significance level F, indicating a risk for potentially life-threatening adverse events, resulted in 13 745 and 91 346 co-occurrences in these subgroups, respectively. Level F co-occurrences accounted for nearly half (1.7%) of the overall number of high risk drug-phenotype co-occurrences in the Medicare > = 65 group, and more than one quarter (1.4%) in the Medicaid 40–64 group.

As described in the Methods section, inclusion of codeine prescriptions in the analysis of clinical significance was deemed problematic, as it might result in overestimations caused by the presumably wider application of codeine as a cough medicine instead of as an analgesic. The inclusion of codeine in this final step of the analysis increased the number of DPWG class C to F co-occurrences by an additional 0.3% to 0.4% for each dataset and age group. Similarly, CPIC level A co-occurrences are increased by an additional 0.3% to 0.4% as well.

Full data on estimation results excluding and including codeine can be found in S7 Table.

## Discussion

This study was motivated by the observation that current data on potential return on investment for pre-emptive pharmacogenomics testing is either not detailed enough in terms of demographics or limited to academic healthcare environments. The results suggest that a significant portion of the population will be exposed to one or more PGx drugs and that considerable variation exists in the PGx drugs that are most frequently prescribed depending on age and insurance. This variation extends to the proportion of individuals for which pre-emptive testing could be used to optimize a multitude of medications and the estimated fraction of high-risk drug-phenotype co-occurrences.

Incident users of two or more PGx drugs ranged between 9% and 33% of patients of age ≥ 14. Older patients enrolled in Medicare (age > = 65) or Medicaid (age 40–64) had the highest incidence of PGx drug use, with approximately half of the patients receiving at least one PGx drug and one fourth to one third of patients receiving two or more PGx drugs. Furthermore, these two subpopulations showed the highest estimated fraction of high-risk drug-phenotype co-occurrences, which pose a risk for inducing significant adverse drug events that are potentially preventable by pre-emptive PGx testing. When interpreting these results, it is important to bear in mind that we report here estimated frequencies of drug-phenotype co-occurrences and not estimated frequencies of adverse drug events. Estimating the actual frequency of adverse drug events would require the inclusion of risk measures in the calculation that describe the actual risk for developing a certain adverse drug reaction in the presence of a certain phenotype, which was beyond the scope of this work. It is also important to note that our results are prone to underestimate the number of actual high-risk drug-phenotype-co-occurrence, since several drug substances with high exposure and high clinical significance (in particular warfarin and simvastatin) were not included in this analysis, as they could not be classified according to the DPWG classification scheme.

We used a conservative approach for this analysis, focusing on incident use of a core list of drugs associated with a small number of well-researched pharmacogenes where testing is feasible with common pharmacogenomic assays. Including genes that are associated with high quality evidence, but are not included in the majority of pharmacogenomic testing panels (HLA-B and IFNL3), or genes with comparatively weak evidence (F5) that are also rarely included in panels, would further increase the percentage of patients exposed to medications with pharmacogenomic recommendations. However, we wanted our results to reflect the current technological capabilities and therefore chose to exclude these genes from our analysis.

The drug substances considered in this analysis were selected based on clinical PGx dosing guidelines authored by two different consortia: the DPWG from the Netherlands, and the CPIC which is primarily composed of members from the USA. It is worth mentioning that, besides clinical evidence, differences in the US and European healthcare systems, such as the prescription drug market or national prescribing practices, may have influenced the consortia’s selection of drug substances for their guidelines. A prominent example for this is the anticoagulant warfarin, which is commonly prescribed in the USA (and subject to a CPIC guideline) whereas in Europe phenprocoumon (covered by a DPWG guideline) is mostly used instead. Furthermore, there are several drug substances covered by the DPWG guidelines that are not yet subject to a CPIC guideline but are listed as “high-priority” (level A) gene-drug combinations, such as UGT1A1 –irinotecan or CYP2D6 –tamoxifen. For this analysis, we decided to unify the DPWG and CPIC list to provide a complementary view of PGx drugs.

This study focused on incident use instead of prevalent use. Focusing on prevalent use would make sense in cases of drug therapy optimization based on PGx markers. Such optimization of ongoing treatments might be especially useful in cases where pharmacogenomic markers confer a long-term risk of adverse events (e.g., risk of thrombosis when taking oral contraceptives [15]), biomarkers of ADE risk do not exist, or in patients with multiple morbidities and polypharmacy, where potential side-effects of improperly dosed medications might be difficult to associate with their root causes. If prevalent use in such cases would be included in the analysis, the fraction of patients who could benefit from pre-emptive testing would increase.

We evaluated a mixed ‘reactive pre-emptive’ scenario and found that the number of different medications prescribed to a tested patient is considerably increased compared to a purely pre-emptive approach in some of the patient populations. This more targeted approach could result in a better return on investment for PGx testing, but also suffers from some of the difficulties associated with reactive PGx testing (e.g., uncertainty about when to order tests). The feasibility of implementing such a ‘reactive pre-emptive’ approach based on the drug substances covered by DPWG guidelines will be further investigated within the context of the European PGx implementation project “Ubiquitous pharmacogenomics” (U-PGx) in a multi-centred cross-over trial, starting in January 2017 [38].

Panel-based pre-emptive testing has the potential to increase the use of pharmacogenetic data based on its immediate availability, because results might either be available from a previous pre-emptive test or additional benefits from the pre-emptive test might be expected in the future. Additionally, the accessibility of test results to healthcare providers can be facilitated by novel information technologies. This includes expanded use of electronic health records, and innovative methods such as the Medication Safety Code (MSC) [39], in which a patient can carry personal pharmacogenomic data on a pocket-sized card. Besides human readable information on pharmacogenomic risk factors, the MSC card contains a barcode which can be scanned with a mobile device to retrieve patient-specific pharmacogenomic guidelines. In the context of U-PGx, the MSC system will be implemented at several European sites as an auxiliary tool for maximizing the utility of pharmacogenomic data and enabling decision support in a wide variety of care settings.

### Limitations

The data show results for pre-emptive and reactive pre-emptive scenarios that may be of use for health systems with patient populations similar to the included populations. However, some health systems might have very different patient populations from those included. In practice, the impact of pre-emptive pharmacogenomic testing might vary depending on other demographics, ethnicity, clinical conditions, diagnosis for which the medication is prescribed, and medication use in the population to which it is applied. Conducting a sensitivity analysis to assess the impact of variations in allele frequency or ethnic distribution on our results was beyond the scope of this work.

Practical constraints and missing data in the utilized datasets made it necessary to apply a pragmatic and therefore simplified model to assess the potential impact of pre-emptive pharmacogenomic testing, resulting in two main limitations: Firstly, some of the PGx guidelines included in this analysis are restricted to specific indications, patient groups or dosage levels. Examples for this include the CPIC guideline for clopidogrel that only addresses patients undergoing percutaneous coronary intervention, and the guideline for codeine that only applies to analgesic doses of codeine. Due to the unavailability of data on indication in the used datasets, our results on incident PGx drug prescriptions do not fully reflect these restrictions.

Secondly, the partly incomplete coverage of ethnicity in the used datasets made it necessary to infer the estimated distribution of ethnic subgroups in the different insurance populations from other publicly available demographic datasets. Thus, the estimation on the prevalence of high risk drug-phenotype co-occurrences does not account for any differences in the prevalence of medication use between different ethnic subpopulations that may result from ethnic predispositions to certain health conditions. These limitations of our study emphasize the importance of the availability of comprehensive data on medication use that, besides data on age and gender, also captures additional parameters, such as ethnicity, diagnosis, and dosage amount.

Future work should also take into account that costs of genetic testing and adverse drug events might vary widely between different institutions and regions. This paper sets the stage of the broader issue of economic implications of PGx testing. However, providing more than speculative estimates is not possible because those studies are complicated and time consuming to conduct. We do plan to develop such studies but that is beyond the scope of the current investigation. We think a promising approach to addressing these issues is to enable a broad range of stakeholders and interested researchers to collaborate on extending the analysis. Towards this goal, we have created a new research study within the Observational Health Data Sciences and Informatics (OHDSI) collaborative [40]. The OHDSI program is a multi-stakeholder, interdisciplinary collaborative with the goal of bringing out the value of health data through large-scale, open source analytics. OHDSI has established an international network of researchers and observational health databases with a central coordinating center housed at Columbia University, New York, USA. Immediate next steps are to generate statistics on genes associated with PGx drugs. Furthermore, we plan to extend the study to cover regions outside the United States.

Finally, the evidence and strength of different pharmacogenomic treatment recommendations varies greatly and is constantly changing as new data are generated by the scientific community; implementers might deem only a subset of the recommendations from CPIC and DPWG suitable for utilisation in clinical practice.

## Conclusions

To our knowledge, this is the first analysis to use large claims datasets to examine the potential for pre-emptive pharmacogenomics testing by examining the incidence of exposure to multiple PGx drugs in different patient populations. However, deriving incidence data is only the first step toward a detailed health economic analysis that predicts the impact of different pre-emptive PGx testing scenarios in different regions and care settings on the quality of care, total costs and cost savings. While this study is not a formal cost-effectiveness analysis, it provides the basis for determining key parameters that are required to estimate the economic implications of implementing the various testing strategies. A formal cost-effectiveness analysis needs to consider not only the incidence of use of these medications, but would also need to quantify risks and benefits associated with those therapies that would be more efficiently used with pharmacogenomic information. This would include not prescribing medications that may not be metabolised into active medications and also preventing debilitating adverse drug reactions and drug-drug interactions.

## Supporting Information

S1 Table Punnett squares. (XLSX)

S2 Table Accumulation of phenotypes. (XLSX)

S3 Table Detailed claims data statistics. (XLSX)

S4 Table Drug-phenotype co-occurrence (CCAE). (XLSX)

S5 Table Drug-phenotype co-occurrence (Medicaid). (XLSX)

S6 Table Drug-phenotype co-occurrence (Medicare). (XLSX)

S7 Table Drug-phenotype co-occurrence overall results. (XLSX)

S1 Text Supplementary methods. (DOCX)
