# Supplementary material for: Incidence of Exposure of Patients in the United States to Multiple Drugs for Which Pharmacogenomic Guidelines Are Available
Source: PLoS One. 2016 Oct 20;11(10):e0164972. doi: 10.1371/journal.pone.0164972 (PMC5072717; doi:10.1371/journal.pone.0164972)
Supplement: S1 Text — (DOCX) [file pone.0164972.s008.docx]

# Supplemental material: Supplementary Methods

## Overview of supplementary tables

Table 1: Overview of supplementary tables

| **File name** | **Description** |
| --- | --- |
| S1 Table – Punnett squares | Contains the **compiled haplotype frequencies** for all genes that were included in the analysis and the **Punnett squares** that were used for calculating the frequency of haplotype combinations. |
| S2 Table – Accumulation of phenotypes | Contains the color-coded **assignments of diplotypes to the drug-metabolizing phenotype classification** (EM – Extensive metabolizer, UM – Ultrarapid metabolizer, IM – Intermediate metabolizer, PM – Poor metabolizer) for each gene, where applicable. These assignments are used to calculate the estimated **overall frequency of drug-metabolizing phenotypes per gene and population group** on the first sheet (“Overview”). |
| S3 Table – Detailed claims data statistics | Contains the detailed statistics on incident claims for PGx drugs within the selected four-year period (1/1/2009 – 12/31/2012) for each dataset and age group. |
| S4 Table – Drug-phenotype co-occurrence (CCAE) | **First sheet:** Contains the assignments of diplotypes to the clinical significance classification by the DPWG (see Table 2) for each phenotype-drug substance pair. Contains the overall statistics on high risk drug-phenotype co-occurrences for the respective dataset, per age group.  **Second sheet:** Contains the estimated number of patients in the respective dataset that are EMs, IMs, PMs or UMs per age group and gene.  **Other sheets:** Contain the estimations on drug-phenotype co-occurrences for each gene. |
| S5 Table – Drug-phenotype co-occurrence (Medicaid) |  |
| S6 Table – Drug-phenotype co-occurrence (Medicare) |  |
| S7 Table - Drug-phenotype co-occurrence overall results | Contains the overall result tables for the estimation on drug-phenotype co-occurrences for two different scenarios: including codeine (second sheet) and excluding codeine (first sheet). |

## Additional information on the process of estimating the number of high risk drug-phenotype co-occurrences in the investigated patient cohorts

### Example: Calculating the frequency of diplotypes via Punnett squares

For the gene CYP2C19, 8 haplotypes (*1-*8, *17), resulting in 64 possible haplotype combinations (diplotypes), were considered. The estimated haplotype frequencies in Caucasians, African Americans and Asians were extracted from the Supplementary material of the CPIC guideline for Citalopram and CYP2C19 (1). For example, CYP2C19*2 and CYP2C19*3 are both loss-of-function alleles with a reported frequency of ~0.146 and ~0.006 in Caucasians. In the Punnett square, the probability for all 64 possible CYP2C19 diplotypes was calculated by multiplying the respective haplotype frequencies. Thus, the probability of having a CYP2C19*2/*3 diplotype is calculated as follows: 0.146*0.006=0.000876. The probability of having a CYP2C19*3/*2 diplotype is calculated the same way. Note, that the probability of having a CYP2C19*2/*3 OR a CYP2C19*3/*2 diplotype would therefore be calculated as follows: 2*0.146*0.006=0.00175.

### Inclusion of drug substances

Table 2 gives an overview about which drug substances were included in our estimation of the number of high risk drug-phenotype co-occurrences and lists the reasons for including / excluding drugs. All drug substances that could be assigned a clinical significance level C-F according to the DPWG guidelines were included, except for codeine. It was decided to remove codeine from the main results because of its widespread use as a low-dosed cough medicine, whereas PGx guidelines generally apply to higher, analgesic dosages of codeine. A sufficiently reliable distinction of low-dose vs high-dose regimen of codeine was not deemed feasible with the datasets used. To quantify the impact of this decision, the calculations were nevertheless conducted for both scenarios (see supplementary material Table S7 – Drug-phenotype co-occurrence overall results).

Drug substances with a clinical significance level A or B were excluded. Drug substances that were not assigned a clinical significance level by the DPWG were excluded.

Table 2: Overview of drug substances that are included in the estimation of high risk drug-phenotype co-occurrences

| **Drug substance** | **Included?** | **Reason** |
| --- | --- | --- |
| Glimepiride | Yes | Clinical significance C-F |
| Phenytoin | Yes | Clinical significance C-F |
| Clopidogrel | Yes | Clinical significance C-F |
| Sertraline | Yes | Clinical significance C-F |
| Amitriptyline | Yes | Clinical significance C-F |
| Clomipramine | Yes | Clinical significance C-F |
| Doxepin | Yes | Clinical significance C-F |
| Imipramine | Yes | Clinical significance C-F |
| Aripiprazole | Yes | Clinical significance C-F |
| Haloperidol | Yes | Clinical significance C-F |
| Metoprolol | Yes | Clinical significance C-F |
| Nortriptyline | Yes | Clinical significance C-F |
| Paroxetine | Yes | Clinical significance C-F |
| Propafenone | Yes | Clinical significance C-F |
| Risperidone | Yes | Clinical significance C-F |
| Tamoxifen | Yes | Clinical significance C-F |
| Tramadol | Yes | Clinical significance C-F |
| Venlafaxine | Yes | Clinical significance C-F |
| 6-Mercaptopurine | Yes | Clinical significance C-F |
| Azathioprine | Yes | Clinical significance C-F |
| Thioguanine | Yes | Clinical significance C-F |
| Codeine | No | Please see text |
| Voriconazole | No | Clinical significance A or B |
| Citalopram | No | Clinical significance A or B |
| Escitalopram | No | Clinical significance A or B |
| Omeprazole | No | Clinical significance A or B |
| Pantoprazole | No | Clinical significance A or B |
| Esomeprazole | No | Clinical significance A or B |
| Lansoprazole | No | Clinical significance A or B |
| Rabeprazole | No | Clinical significance A or B |
| Mirtazapine | No | Clinical significance A or B |
| Flecainide | No | Clinical significance A or B |
| Oxycodone | No | Clinical significance A or B |
| Olanzapine | No | Clinical significance A or B |
| Atomoxetine | No | Clinical significance A or B |
| Carvedilol | No | Clinical significance A or B |
| Clozapine | No | Clinical significance A or B |
| Duloxetine | No | Clinical significance A or B |
| Tolbutamide | No | Clinical significance A or B |
| Trimipramine | No | Clinical significance N/A |
| Warfarin | No | Clinical significance N/A |
| Desipramine | No | Clinical significance N/A |
| Tacrolimus | No | Clinical significance N/A |
| Capecitabine | No | Clinical significance N/A |
| Fluorouracil | No | Clinical significance N/A |
| Simvastatin | No | Clinical significance N/A |
| Irinotecan | No | Clinical significance N/A |

### Ethnic distribution in the CCAE and Medicare datasets

The CCAE and Medicare Supplemental datasets did not contain data on population groups. Demographic statistics on these insurance populations were derived from an external source (i.e. the Kaiser Family Foundation [KFF], a US non-profit organization that publishes reports on health care issues) to estimate the distribution in the investigated cohorts (2,3). For CCAE, KFF reported statistics on ethnicity for the age groups 0-18 and >=19 for the year 2011. To take account for the different definition of age groups in our analysis, we adjusted these statistics as follows: For the age groups 0-13 and 40-64 the reported KFF statistics for the age groups 0-18 and >=19 were used, respectively. For the age group 14-39, we used the following calculation for an adjusted approximation of the distribution: 0.2 * (age group 0-18) + 0.8 * (age group >=19). For Medicare, KFF statistics for the year 2014 were applied directly, since the majority (>90%) of Medicare enrolees is aged >=65.

### Example: Estimating the number of CYP2C19 poor metabolizer / codeine prescription co-occurrences for the Medicare insurance group

Calculating the probability that a PGx drug is prescribed to a patient who has a drug metabolizing phenotype that puts the patient at risk for developing an adverse drug reaction if the drug is prescribed in standard dosage$p\left( \mathrm{Rx} \right| risk phenotype)$ was performed as follows:

$$p\left( Rx \right| risk phenotype)= p\left( Rx \right)\times p\left( risk phenotype | ethnicity \right) \times p(ethnicity)$$

where p(Rx) is the probability that a patient is prescribed a PGx drug in the observed time frame (see supplementary table S3), p(risk phenotype | ethnicity) is the estimated prevalence of risk phenotypes relevant to the respective PGx drug in the respective population group (see supplementary table S2) and p(ethnicity) is the prevalence of the ethnic population group in the respective dataset and age group (see supplementary tables S4-7).

According to the KFF statistics (see above), the percentage of “White” / Caucasian Medicare insurance holders $p(ethnicity)$ was estimated to be 83.5%. The percentage of CYP2C19 poor metabolizers among “White” / Caucasian patients $p\left( risk phenotype \right| ethnicity)$ was estimated to be 2.56%. According to the results of our drug prescription analysis, the probability that a Medicare insurance holder is prescribed codeine in the observed time frame is 5.1%. Therefore, the probability that a Caucasian Medicare insurance holder who is a CYP2C19 poor metabolizer is prescribed codeine in the observed time frame is calculated by: 0.835 x 0.0256 x 0.051 = 0.0011.

This calculation was equally performed for the Asian population group (0.05 x 0.14 x 0.051 = 0.00036) and the African American / Black population group (0.11 x 0.035 x 0.051 = 0.0002). Multiplying the sum of these probabilities (0.0017) with the overall number of patients in the Medicare cohort (n = 5 429 266) gives the number of CYP2C19 poor metabolizer / codeine prescription co-occurrences for the Medicare insurance group (n = 9229).

These calculations were equally conducted for all other drug-phenotype combinations and summed up to give the overall number of high risk drug-phenotype co-occurrences in the Medicare subpopulation.

### References

1. Hicks JK, Bishop JR, Sangkuhl K, Müller DJ, Ji Y, Leckband SG, et al. Clinical Pharmacogenetics Implementation Consortium (CPIC) Guideline for CYP2D6 and CYP2C19 Genotypes and Dosing of Selective Serotonin Reuptake Inhibitors. Clin Pharmacol Ther. 2015 Aug;98(2):127–34.

2. The Kaiser Family Foundation: Medicare beneficiaries by race and ethnicity [Internet]. 2016 [cited 2016 Apr 27]. Available from: http://kff.org/report-section/profile-of-medicare-beneficiaries-by-race-and-ethnicity-chartpack/

3. The Kaiser Family Foundation: Health coverage by race and ethnicity [Internet]. 2016 [cited 2016 Apr 27]. Available from: http://kff.org/disparities-policy/issue-brief/health-coverage-by-race-and-ethnicity-the-potential-impact-of-the-affordable-care-act/
